# Safety, tolerability, pharmacokinetics, and immunogenicity of JMB2002–an antibody against COVID-19: a phase 1 clinical trial in healthy Chinese adults

**DOI:** 10.1186/s12879-023-08341-6

**Published:** 2023-06-27

**Authors:** Guiling Chen, Ying Zhang, Kaiqi Wu, Tinghan Jin, Conggao Peng, Qi Jiang, Wenjuan Tian, Zhong Chen, Zhenwei Shen, Guoping Sheng

**Affiliations:** 1 Institute of Pharmacology and Toxicology, College of Pharmaceutical Sciences, Zhejiang University, Hangzhou, China; 2 Shulan (Hangzhou) Hospital Affiliated to Zhejiang Shuren University Shulan International Medical College, Hangzhou, China; 3 Shanghai Jemincare Pharmaceutical Co., Ltd., Shanghai, China; 4 Zhejiang Shuren University, Hangzhou, China

**Keywords:** JMB2002, SARS-CoV-2, COVID-19

## Abstract

**Background:**

The emergence of Severe Acute Respiratory Syndrome Coronavirus 2 (SARS-CoV-2) and subsequent Coronavirus Disease 2019 (COVID-19) pandemic has resulted in a significant global public health burden, leading to an urgent need for effective therapeutic strategies. Monoclonal antibodies (mAbs) are a potentially effective therapeutic option. We identified a potent antibody JMB2002 against the SARS-CoV-2 receptor binding domain. JMB2002 has demonstrated therapeutic efficacy in a SARS-CoV-2 infected rhesus macaque model.

**Methods:**

We conducted a randomized, double-blind, phase 1 trial to evaluate the JMB2002’s safety, tolerability, pharmacokinetics, and immunogenicity in healthy Chinese adults. Participants were randomly assigned to one of four cohorts with sequential dose, administrated intravenously with JMB2002 or placebo, and followed up for 85 ± 5 days.

**Results:**

40 participants were recruited and completed in the study. Eight (25.0%) participants experienced 13 treatment emergent adverse events (TEAEs) that were drug-related. No serious adverse events (SAEs), dose limiting events (DLTs), or adverse events of special interest (AESIs), such as infusion related/allergic reactions, were observed, and no drop out due to adverse events (AEs) occurred. There was no significant safety difference observed between JMB2002 and the placebo, suggesting it was well tolerated. The AUC_0−∞_, AUC_0 − t_ of JMB2002 infusion increased dose-dependently from 5 mg/kg to 50 mg/kg while there is also a linear trend between doses and C_max_.

**Conclusion:**

Therefore, JMB2002 was well tolerated after administration of a single dose in the range of 5 mg/kg to 50 mg/kg in healthy Chinese adults.

**Trial registration:**

ChiCTR2100042150 at https://www.chictr.org.cn/searchproj.aspx (14/01/2021).

## Background

In late 2019, the novel beta corona virus SARS-CoV-2 emerged from an animal reservoir into humans, causing an acute respiratory infection known as COVID-19 [1, 2]. Despite the effect of many strategies [3], such as vaccination, masking, social distancing and community lockdown, have been applied worldwide to prevent the virus spreading, it has infected more than 281 million people globally and caused more than 5.4 million deaths as of December 2021 [4]. One major challenge against the COVID-19 pandemic was the high mutation rate of SARS-CoV-2 virus, which led to enhanced transmission and antibody evasion. Within two years, several major variants, such as Alpha, Beta, Delta, and Omicron variants, have emerged with characterized mutations in the spike protein that affects the binding affinity of angiotensin-converting enzyme 2 (ACE2) receptor and alters antibody epitopes. Especially, the Omicron variant was recently identified in south Africa in November 2021 and quickly spread over the world. With over 30 mutations in spike protein, this variant has demonstrated high infectivity and strong ability to escaping vaccine-induced immune response or antibodies present in convalescent individuals [5, 6].

One approach to combating pathogen outbreak, such as SARS-CoV-2, would be to use plasma from the convalescent patients [4, 7], e.g., both SARS and Ebola patients benefited from receiving the treatment of convalescent plasma [8, 9]. On the other hand, the monoclonal neutralizing antibodies (NAbs) targeting S protein, particularly the receptor-binding domain (RBD) has potential to be effective in treating SARS-CoV-2 infection [10, 11]. Through the RBD in S protein, SARS-CoV-2 virus directly binds to the angiotensin-converting enzyme 2 (ACE2) receptor of human epithelial cells to facilitate the viral entry into host cells. The structures of human ACE2 and SARS-CoV-2 S protein trimer show that the RBD on the viral S protein S1 subunit directly contacts with human ACE2 [12]. Therefore, the NAbs blocking the ACE2-S protein interaction are expected to prohibit the viral entry. Nevertheless, several candidate NAbs on the pipeline yield minimal efficacy results [11, 13]. More, recent-emerging Omicron variant was totally or partially resistant to neutralization by monoclonal NAbs clinically approved or in development [14, 15].

JMB2002–a human monoclonal antibody specifically binds to the RBD in S protein of SARS-CoV-2, was developed by Shanghai JiYu Pharmaceutical Technology Co. LTD, through a self-created FeiTai platform. It is a fully human IgG1 antibody containing N297A mutation in its constant region to attenuate Fc function [16]. There is a theoretical risk of Fc effector function associated with antibody-dependent enhancement (ADE). This modification aimed to circumvent this risk, reduce or eliminate its in vitro ADE activity, and ensure its high safety in vivo [17]. In subsequent studies with other neutralizing antibodies, no ADE was reported [18]. In silico analysis, along with a comparable somatic hypermutation rate, predicted its low immunogenicity compared to some marketed therapeutic antibodies such as Etesevimab or Imdevimab [17]. It has demonstrated high potency in neutralizing SARS-CoV-2 virus, by blocking its binding to the ACE2 receptor [16], thereby the viral entry to the host cells, and showed potent prophylactic and therapeutic efficacy in rhesus macaques model [17]. Most importantly, JMB2002 could bind to a broad range of SARS-CoV-2 RBD variants with high affinity [16], indicating that this mAb has broad-spectrum activity against SARS-CoV-2 strains, including the recent-emerging omicron variants [19, 20]. In contrast to other characterized antibodies, our in vitro study demonstrated that JMB2002 bound to Omicron spike protein in a similar kinetic as its binding to that of wild type (equilibrium dissociation constant K_D_=0.47 nM), inhibited the binding of ACE2 to the Omicron spike protein, and blocked the entry of Omicron pseudo virus into human ACE2 expressing cells [5]. The pseudovirus neutralization test proved that it has high affinity and neutralization ability to SARS-CoV-2 α, β, γ and omicron strains, including BA.1, BA.2 [5, 21]. Further, preclinical studies, including toxicology, pharmacodynamics and pharmacokinetic data, showed that JMB2002 had high safety, with no effect on the function of cardiovascular system, respiratory system, or central nervous system [17] (data not shown), and support its eligibility for clinical trial. Here, we evaluated its safety, tolerability, pharmacokinetics, and immunogenicity in a randomized double-blind, placebo-controlled phase I clinical trial.

## Methods

### Study design and participants

This phase 1 clinical trial was a randomized double-blind, placebo-controlled study. Forty enrolled participants were randomly and sequentially allocated (4:1) to four escalation dose cohorts of 5, 15, 30 to 50 mg/kg.

This study was conducted in accordance with the principles of Declaration of Helsinki and Good Clinical Practice. The protocol and informed consent were confirmed by the Clinical Trial Ethics Committee of our Hospital. All participants signed informed consents before receiving the screening. This trial was registered with Chinese Clinical Trial Registry, ChiCTR2100042150.

### Inclusion and exclusion criteria

Participants aged 18 ~ 45 years in good health were eligible to enrolled. Exclusion criteria included those confirmed cases of previous coronavirus infection or carriers, suspected coronavirus disease, and high-risk exposure within 14 days. A complete list of inclusion and exclusion criteria was written in the study protocol ChiCTR2100042150 at Chinese Clinical Trial Registry(https://www.chictr.org.cn/searchproj.aspx).

### Randomization and masking

The allocation and randomization numbers were generated by SAS 9.4, through the interactive web response system. After participants signed informed consent, they were assigned a screening number for screening process. Every eligible participant who fully met the screening criteria was randomized on Day-1 after assigned a randomization number in ascending order of the screening number by gender respectively. All investigators, participants, study coordinators and related personnel, and the sponsor were masked to allocation. JMB2002 (WuXi Biologics) and placebo, both were colorless to light yellow and transparent to micro-opalescent liquid, were identical in the appearance, viscosity and color.

### Dose escalation

Based on preclinical studies, this study applied 4 dose levels, from 5, 15, 30 to 50 mg/kg. The study started from the lowest dose group and proceeded, to the next dose level only after safety assessment was confirmed not reaching the below-listed criteria for dose escalation termination. For the safety assessment, dose limit toxicity (DLT) was observed for at least 7 days. Blindness was kept when assessing the data of all subjects of the current dose group. No subject participated in two or more dose groups.

The dose escalation termination criteria were: (1) more than 1/2 participants had drug-related adverse events (AEs) ≥ grade 2 (CTCAE 5.0), or (2) more than 1/4 participants had drug-related AEs ≥ grade 3, or (3) 1 drug-related serious adverse event (SAE). Termination criteria for sentinels was that at least 1 participant had drug-related AEs ≥ grade 3 or 1 drug-related SAE. All AEs were graded using CTCAEv5.0 (Common Terminology Criteria for Adverse Event) and coded with MedDRA23.1 (Medical Dictionary for Regulatory Activities).

### Study procedures

Both JMB2002 and placebo were diluted in a 0.9% sodium chloride (250 mL), and then were perfused intravenously for 60 min. First 10 participants were randomized (4:1) to receive 5 mg/kg of JMB2002 or placebo in cohort 1, and monitored for adverse events. After a successful safety evaluation on Day 7, the cohort 2 was proceeded with another 10 randomly-selected participants receiving 15 mg/kg of JMB2002 or placebo (4:1), and so on. Particularly in cohort 3 (30 mg/kg) and 4 (50 mg/kg), two sentinels were first randomly selected and assigned (1:1) to receive JMB2002 or placebo respectively; and only if there were no drug-related AEs ≥ graded 3 after day 1, the remaining 8 participants were proceeded.

Vital signs, such as blood pressure, pulse, respiration and ear temperature, were taken at 0 h as baseline, then 1, 3, 8, 24, 48, and 72 h after administration and on days 8, 15, 29, 43, 57, and 85 or early termination day. Physical examination, including skin, mucous membrane, lymph node and so on, was taken on days 8, 29,57 and 85 or early termination day. Laboratory examination, including blood routine, urine routine, blood biochemistry and coagulation function, and 12-lead electrocardiogram were carried out on days 4,8,29,57 and 85 or early termination day.

#### Outcomes

The primary outcome was the safety and tolerability of the JMB2002 in healthy Chinese adults, which was quantified by the number and proportion of treatment emergent adverse events (TEAEs). Any anomaly in the exam results and occurred adverse medical events were also recorded. Secondary outcomes were pharmacokinetic characteristics and immunogenicity. Pharmacokinetic parameters were calculated and analyzed using non-compartmental analysis (Phoenix WinNonlin version 6.4 or above), by measuring the concentration of JMB2002 in serum of participants at 0 h as baseline, then 1, 3, 8, 24, 48 and 72 h, and on days 8, 15, 29, 43, 57 and 85 after receiving a single dose of JMB2002 by intravenous infusion. The area under the serum concentration–time curve from time zero to the time of the last quantifiable concentration (AUC_0–t_) or time to infinity (AUC_0−∞_), maximum concentration (C_max_), elimination half-life (t_1/2_), volume of distribution (V_z_), plasma clearance (CL_z_), time to reach Cmax (T_max_), the terminal phase elimination rate constant (λ_z_), and mean residence time (MRT), were calculated accordingly [16]. Dose linearity and proportionality of C_max_, AUC_0–t_, or AUC_0−∞_ respectively was performed after log-transformation, using a power model first described by Gough et al. [20, 21]. Serum anti-JMB2002 antibody titers were measured by ELISA (Shanghai TriApex Biotechnology Co., Ltd) to determine the anti-drug antibody (ADA) at 5 time points to assess immunogenicity: 0, 15, 29, 57 and 85 days after administration.

### Statistical analysis

All statistical analyses were completed using SAS 9.4. Quantitative variables were described with number, arithmetic mean, standard deviation (SD), median, Q1, Q3, minimum and maximum. Pharmacokinetic variables were additionally described with coefficient of variation and geometric mean. Count variable was described with frequency and percentage. Unless otherwise stated, all statistical tests were two-sided with α = 0.05, and two-sided 95% confidence intervals were calculated. If the P value was ≥ 0.001, it was rounded to 3 decimal places; if the P value was < 0.001, it was reported as “<0.001”.

## Results

### Baseline characteristics

**Fig. 1:**
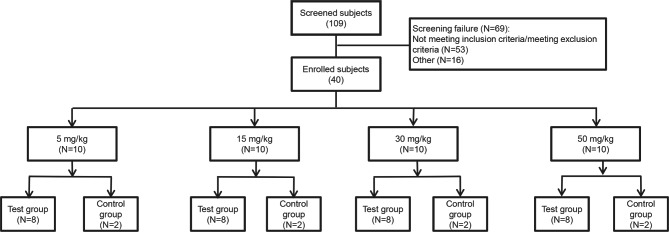
Scheme of participant selection for the clinical trial

A total of 109 screened subjects, 40 participants were eligible and enrolled in the study. Ten participants were randomly assigned into 5, 15, 30, and 50 mg/kg dose group respectively, of which 8 received JMB2002 and 2 received placebo (Fig. 1). All 40 participants complied to the planned dosage, without dropout.

The baseline characteristics and demographic information of 40 participants are listed in (Table 1). The mean age (SD) of participants was 29.7 (6.50) years (range: 21, 45 years) with 63.6 (8.3) kg (range: 47.7, 79.0 kg) mean weight. Demographics and baseline characteristics were well balanced between the test group and the control group at each dose level (Table 1).

**Table 1:** Participants characteristics

	5 m/kg		15 mg/kg		30 mg/kg			50 mg/kg			Total				
	Test group	Control group	Test group	Control group		Test group	Control group		Test group	Control group		Test group	Control group		Total
	(N = 8)	(N = 2)	(N = 8)	(N = 2)		(N = 8)	(N = 2)		(N = 8)	(N = 2)		(N = 32)	(N = 8)		(N = 40)
**Age (years)**															
Mean (range)	34.3 (27, 45)	34.0 (33, 35)	32.0 (33, 35)	36.0 (31, 41)		27.9 (23, 34)	27.5 (27, 28)		24.4 (21, 29)	22.5 (21, 24)		29.6 (21, 45)	30.0 (21, 41)		29.7 (21, 45)
**Sex [n(%)]**															
Male	6 (75.0)	1 (50.0)	6 (75.0)	1 (50.0)		5 (62.5)	1 (50.0)		7 (87.5)	1 (50.0)		24 (75.0)	4 (50.0)		28 (70.0)
Female	2 (25.0)	1 (50.0)	2 (25.0)	1 (50.0)		3 (37.5)	1 (50.0)		1 (12.5)	1 (50.0)		8 (25.0)	4 (50.0)		12 (30.0)
**Nationality [n(%)]**															
Han nationality	7 (87.5)	2 (100.0)	8 (100.0)	2 (100.0)		7 (87.5)	2 (100.0)		8 (100.0)	2 (100.0)		30 (93.8)	8 (100.0)		38 (95.0)
Other	1 (12.5)	0 (0.0)	0 (0.0)	0 (0.0)		1 (12.5)	0 (0.0)		0 (0.0)	0 (0.0)		2 (6.3)	0 (0.0)		2 (5.0)
**Height (cm)**															
Mean (range)	166.30 (155.0, 180.0)	166.35 (158.5, 174.2)	165.41 (156.4, 174.9)	164.80 (158.0, 171.6)		164.71 (154.3, 171.2)	160.00 (158.3, 161.7)		172.49 (165.7, 181.8)	166.50 (164.2, 168.8)		167.23 (154.3, 181.8)	164.41 (158.0, 174.2)		166.67 (174.2, 181.8)
**Weight (kg)**															
Mean (range)	66.09 (50.3, 76.3)	56.45 (47.7, 65.2)	62.18 (47.9, 74.2)	69.00 (68.7, 69.3)		61.34 (49.6, 74.0)	60.35 (53.1, 67.6)		66.18 (55.5, 79.0)	63.90 (63.7, 64.1)		63.94 (47.9, 79.0)	62.43 (47.7, 69.3)		63.64 (47.7, 69.3)
**BMI (kg/m** ^**2**^ **)**															
Mean (range)	23.8 (21, 28)	20.0 (21, 28)	22.6 (20, 27)	25.5 (23, 28)		22.8 (19, 27)	23.5 (20, 27)		22.4 (19, 28)	23.0 (22, 24)		22.9 (19, 28)	23.0 (19, 28)		22.9 (19, 28)

The percentage was calculated based on the number of subjects enrolled in each dose group

### Safety

All the participants received a single dose of JMB2002 over the range of 5 to 50 mg/kg or placebo by intravenous infusion. Within the entire duration of the study, a total of 17 (42.5%) participants developed 27 cases of TEAEs, including 13 (40.6%) in the test groups and 4 (50%) in the control group. Among them, 11 (27.5%) participants developed 17 drug-related TEAEs, including 8 (25.0%) in the test groups and 3 (37.5%) in control group (Table 2). No significant difference observed between JMB2002 and the placebo groups in the frequencies of TEAEs or drug-related TEAEs, suggesting that JMB2002 was well tolerated.

**Table 2:** Treatment emergent adverse events after a single-dose infusion of JMB2002

	5 mg/kg		15 mg/kg		30 mg/kg		50 mg/kg		Total		
	Test group	Control group	Test group	Control group	Test group	Control group	Test group	Control group	Test group	Control group	Total
	(N = 8)	(N = 2)	(N = 8)	(N = 2)	(N = 8)	(N = 2)	(N = 8)	(N = 2)	(N = 32)	(N = 8)	(N = 40)
All TEAEs	2 (25.0)	2 (100.0)	4 (50.0)	1 (50.0)	3 (37.5)	0 (0.0)	4 (50.0)	1 (50.0)	13 (40.6)	4 (50.0)	17 (42.5)
Drug-related TEAEs	1 (12.5)	2 (100.0)	2 (25.0)	0 (0.0)	1 (12.5)	0 (0.0)	4 (50.0)	1 (50.0)	8 (25.0)	3 (37.5)	11 (27.5)
Drug-related Grade > = 3 TEAEs	0 (0.0)	0 (0.0)	0 (0.0)	1 (50.0)	0 (0.0)	0 (0.0)	0 (0.0)	0 (0.0)	0 (0.0)	0 (0.0)	0 (0.0)

Abbreviations: TRAEs, Treatment related adverse events

No participants developed grade ≥ 3 TEAEs, grade ≥ 3 drug-related TEAEs, SAEs or drug-related SAEs. Except 1 participant in the test group at 50 mg/kg dose level developed 2 grade 2 drug-related TEAEs, which included “general disorders and administration site conditions/chest discomfort” and “vascular and lymphatic disorders/flushing”, all the other drug-related TEAEs were grade 1. All the participants with TEAEs recovered without sequelae, except for 1 case of “dermatitis” and 1 case of “pharyngalgia” that were determined to be “unlikely related” to the study drug (Table 3). Therefore, JMB2002 was safe and well tolerated in participants following a single dose by intravenous infusion over the range of 5 mg/kg to 50 mg/kg.

**Table 3:** TEAEs summarized by MedDAR system organ, preferred term

system organ classification preferred term	5 mg/kg		15 mg/kg		30 mg/kg		50 mg/kg		Total		Total
	Test group	Control group	Test group	Control group	Test group	Control group	Test group	Control group	Test group	Control group	
	(N = 8)	(N = 2)	(N = 8)	(N = 2)	(N = 8)	(N = 2)	(N = 8)	(N = 2)	(N = 32)	(N = 8)	(N = 40)
	**n (%), event**										
All TEAEs	2 (25.0), 2	2 (100.0), 3	4 (50.0), 5	1 (50.0), 1	3 (37.5), 5	0 (0.0), 0	4 (50.0), 8	1 (50.0), 3	13 (40.6), 20	4 (50.0), 7	17 (42.5), 27
Lab Exams	1 (12.5), 1	1 (50.0), 1	2 (25.0), 3	1 (50.0), 1	1 (12.5), 3	0 (0.0), 0	3 (37.5), 5	0 (0.0), 0	7 (21.9), 12	2 (25.0), 2	9 (22.5), 14
Proteinuria	1 (12.5), 1	0 (0.0), 0	0 (0.0), 0	0 (0.0), 0	0 (0.0), 0	0 (0.0), 0	1 (12.5), 2	0 (0.0), 0	2 (6.3), 3	0 (0.0), 0	2 (5.0), 3
Hyperlipidemia	0 (0.0), 0	0 (0.0), 0	0 (0.0), 0	0 (0.0), 0	1 (12.5), 3	0 (0.0), 0	1 (12.5), 1	0 (0.0), 0	2 (6.3), 4	0 (0.0), 0	2 (5.0), 4
Leukocytosis	0 (0.0), 0	1 (50.0), 1	0 (0.0), 0	0 (0.0), 0	0 (0.0), 0	0 (0.0), 0	0 (0.0), 0	0 (0.0), 0	0 (0.0), 0	1 (12.5), 1	1 (2.5), 1
High ALT	0 (0.0), 0	0 (0.0), 0	1 (12.5), 1	0 (0.0), 0	0 (0.0), 0	0 (0.0), 0	0 (0.0), 0	0 (0.0), 0	1 (3.1), 1	0 (0.0), 0	1 (2.5), 1
Prolonged QT	0 (0.0), 0	0 (0.0), 0	0 (0.0), 0	0 (0.0), 0	0 (0.0), 0	0 (0.0), 0	1 (12.5), 1	0 (0.0), 0	1 (3.1), 1	0 (0.0), 0	1 (2.5), 1
Hypertriglyceridemia	0 (0.0), 0	0 (0.0), 0	0 (0.0), 0	0 (0.0), 0	0 (0.0), 0	0 (0.0), 0	1 (12.5), 1	0 (0.0), 0	1 (3.1), 1	0 (0.0), 0	1 (2.5), 1
HyperCKemia	0 (0.0), 0	0 (0.0), 0	1 (12.5), 1	0 (0.0), 0	0 (0.0), 0	0 (0.0), 0	0 (0.0), 0	0 (0.0), 0	1 (3.1), 1	0 (0.0), 0	1 (2.5), 1
Hypokalemia	0 (0.0), 0	0 (0.0), 0	0 (0.0), 0	1 (50), 1	0 (0.0), 0	0 (0.0), 0	0 (0.0), 0	0 (0.0), 0	0 (0.0), 0	1 (12.5), 1	1 (2.5), 1
Hypoglycemia	0 (0.0), 0	0 (0.0), 0	1 (12.5), 1	0 (0.0), 0	0 (0.0), 0	0 (0.0), 0	0 (0.0), 0	0 (0.0), 0	1 (3.1), 1	0 (0.0), 0	1 (2.5), 1
Respiratory, Thoracic and Mediastinal Disorders	0 (0.0), 0	0 (0.0), 0	2 (25.0), 2	0 (0.0), 0	0 (0.0), 0	0 (0.0), 0	0 (0.0), 0	1 (50.0). 2	2 (6.3), 2	1 (12.5), 2	3 (7.5), 4
Pharyngalgia	0 (0.0), 0	0 (0.0), 0	2 (25.0), 2	0 (0.0), 0	0 (0.0), 0	0 (0.0), 0	0 (0.0), 0	0 (0.0), 0	2 (6.3), 2	0 (0.0), 0	2 (5.0), 2
Epistaxis	0 (0.0), 0	0 (0.0), 0	0 (0.0), 0	0 (0.0), 0	0 (0.0), 0	0 (0.0), 0	0 (0.0), 0	1 (50.0), 2	0 (0.0), 0	1 (12.5), 2	1 (2.5), 2
Skin and subcutaneous tissue diseases	0 (0.0), 0	1 (50.0), 1	0 (0.0), 0	0 (0.0), 0	0 (0.0), 0	0 (0.0), 0	0 (0.0), 0	1 (50.0), 1	0 (0.0), 0	2 (25.0), 2	2 (5.0), 2
Dermatitis	0 (0.0), 0	1 (50.0), 1	0 (0.0), 0	0 (0.0), 0	0 (0.0), 0	0 (0.0), 0	0 (0.0), 0	0 (0.0), 0	0 (0.0), 0	1 (12.5), 1	1 (2.5), 1
Skin rashes	0 (0.0), 0	0 (0.0), 0	0 (0.0), 0	0 (0.0), 0	0 (0.0), 0	0 (0.0), 0	0 (0.0), 0	1 (50.0), 1	0 (0.0), 0	1 (12.5), 1	1 (2.5), 1
Digestive diseases	1 (12.5), 1	0 (0.0), 0	0 (0.0), 0	0 (0.0), 0	1 (12.5), 1	0 (0.0), 0	0 (0.0), 0	0 (0.0), 0	2 (6.3), 2	0 (0.0), 0	2 (5.0), 2
Oral lichen planus	1 (12.5), 1	0 (0.0), 0	0 (0.0), 0	0 (0.0), 0	0 (0.0), 0	0 (0.0), 0	0 (0.0), 0	0 (0.0), 0	1 (3.1), 1	0 (0.0), 0	1 (2.5), 1
Dyspepsia	0 (0.0), 0	0 (0.0), 0	0 (0.0), 0	0 (0.0), 0	1 (12.5), 1	0 (0.0), 0	0 (0.0), 0	0 (0.0), 0	1 (3.1), 1	0 (0.0), 0	1 (2.5), 1

Abbreviations: ALT, alanine aminotransferase; TRAEs, Treatment related adverse events

### Pharmacokinetics

**Fig. 2:**
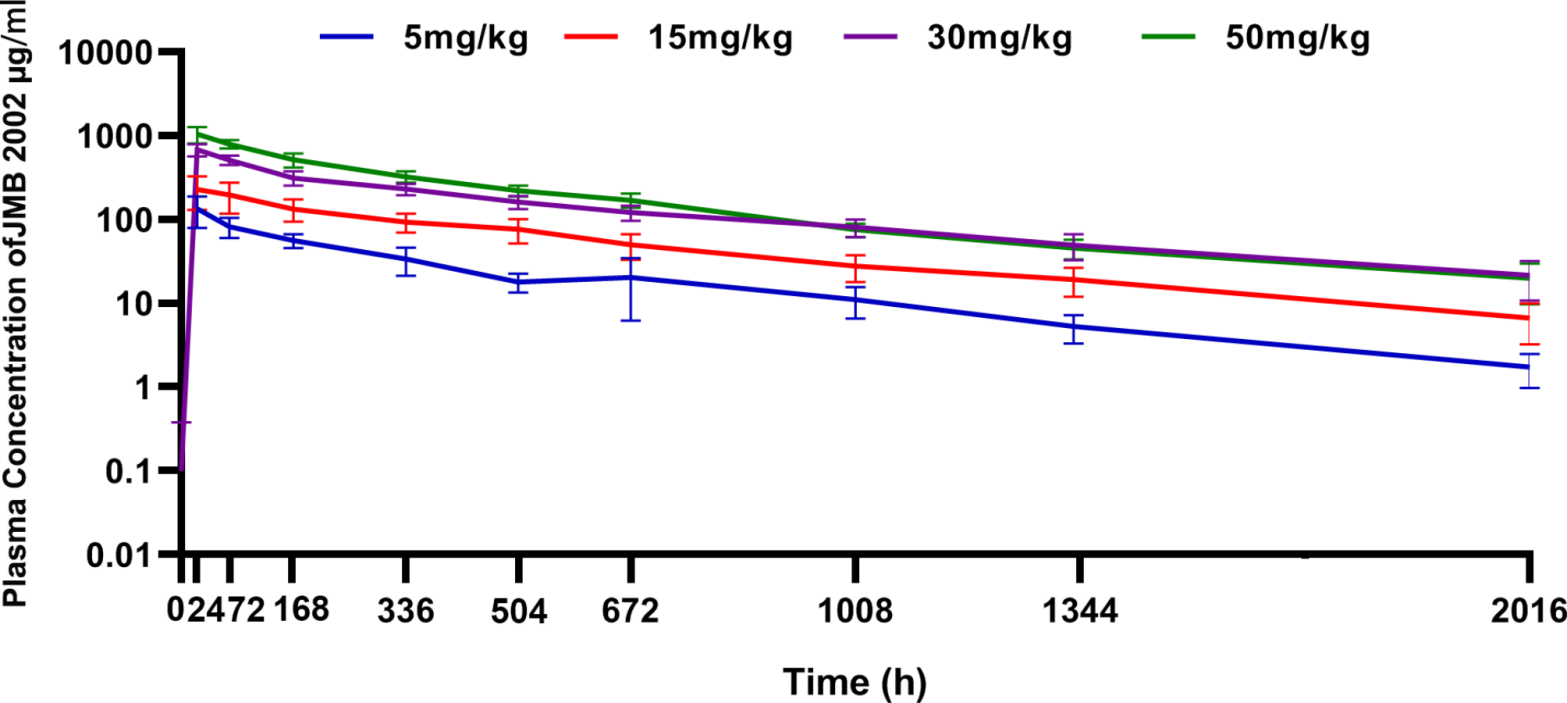
Pharmacokinetic characteristic. Mean plasma concentration-time curves (semi-log scale) after administration in different dose groups

Pharmacokinetic parameters were calculated and analyzed by measuring the serum concentration of the JMB2002 in a serial time course after single intravenous infusion. Plasma concentration plot showed that the plasma JMB2002 concentration increased rapidly after infusion, and the C_max_ increased along with increasing infusion doses over the range of 5 to 50 mg/kg (Fig. 2). Analysis of pharmacokinetic parameters showed that C_max_, AUC_0 − t_, AUC_0−∞_ increased along with increasing infusion doses (Table 4). The C_max_ (mean ± SD) was 141.5 ± 49.4, 310.7 ± 166.8, 841.6 ± 149.6, and 1340.6 ± 226.1 ug/mL in the 5, 15, 30, and 50 mg/kg groups respectively; and the AUC_0 − t_ (mean ± SD) was 39984.0 ± 5594.5, 104043.0 ± 26631.5, 265375.9 ± 43010.3, and 366394.5 ± 45374.6 h*ug/mL respectively. A dose proportionality analysis revealed that AUC_0−∞_, AUC_0 − t_ exhibited linear relationship with the dose of the study drug while there is also a linear trend between dose and C_max_. (Table 5).

**Table 4:** Pharmacokinetic parameters of JMB2002 after a single-dose infusion

		AUC_0 − t_	AUC_0−∞_	C_max_	t_1/2_	V_z_	CL	T_max_	λ_z_	MRT
**Dose level**	**Statistics**	**(**h*ug/mL**)**	**(**h*ug/mL**)**	**(ug/mL)**	**(h)**	**(mL/kg)**	**(mL/h/kg)**	**(h)**	**(1/h)**	**(h)**
**5 mg/kg**	Geomean (%CV)	39654.737 (13.992 )	40615.473 (14.033)	134.827 (34.913)	372.079 (17.274)	66.083 (23.603)	0.123 (13.178)	7.832 (80.801)	0.002 (0.000)	461.147 (21.712)
**15 mg/kg**	Geomean (%CV)	101202.457 (25.597)	105807.749 (25.096 )	282.113 (53.666)	445.476 (23.806)	91.111 (30.126)	0.142 (24.713)	6.353 (144.025 )	0.002 (31.849)	576.623 (21.402)
**30 mg/kg**	Geomean (%CV)	262395.159 (16.207)	278524.504 (18.602 )	831.925 (17.779)	507.997 (20.634)	78.939 (14.041)	0.108 (17.821)	3.130 (142.725)	0.001 (35.635)	616.328 (21.206)
**50 mg/kg**	Geomean (%CV)	363801.487 (12.384 )	378641.010 (12.590)	1322.970 (16.867)	476.539 (22.023)	90.786 (22.960)	0.132 (13.952 )	2.181 (97.418)	0.001 (35.635)	494.439 (23.543)

Values shown are geomeatric mean values (%CV)

**AUC**_**0–t**_, *Area under the concentration-time curve from time zero to the last detectable concentration;***AUC**_**0−∞**_, *Area under the concentration-time curve from time zero to infinity;***C**_**max**_, *Maximum concentration;***t**_**1/2**,_*Elimination half-life;***V**_**z**_, *volume of distribution;***CL**_**z**,_*plasma clearance;***T**_**max**_, *Time to maximum concentration;***λ**_**z**_, *Terminal phase elimination rate constant;***MRT**, *Mean residence time*

**Table 5:** Dose proportionality analysis between pharmacokinetic parameters and JMB2002

PK parameters	Adjusted slope	90% CI	Acceptance range	Dose Proportionality
C_max_	1.022	0.907, 1.137	0.903, 1.097	-
AUC_0-t_	0.998	0.929, 1.067	0.903, 1.097	Linear characteristic
AUC_0-inf_	1.008	0.937, 1.079	0.903, 1.097	Linear characteristic

### Immunogenicity

All 40 participants were followed-up at 5 time points to assess immunogenicity: 0, 15, 29, 57 and 85 days after administration. One participant (2.5%) in the 50 mg/kg dose group was found to have ADA prior to administration (day 0) and tested positive for antibody titers at day 85. The similar results have been reported in other clinical trials [22]. It was concluded that the production of ADA was not due to JMB2002 administration. It suggested that the JMB2002 was low risk of immunogenicity. No further testing neutralizing antibody was performed.

## Discussion

The primary objective of this study was to evaluate the safety and tolerability of single dose of JMB2002 by intravenous infusion in healthy participants. The incidence of drug-related TEAEs at 50 mg/kg dose level was higher than that at other dose levels, suggesting that increased JMB2002 may increase the safety risk. Nevertheless, it remained tolerable in all the participants, with only 1 grade 2 and no grade ≥ 3 or worser TEAEs.

Our results are comparable to similar drugs reported in previous literatures. In a phase I clinical study, Etesevimab (also known as CB6, JS016, LY3832479, or LY-CoV016), a recombinant neutralizing human IgG1 monoclonal antibody developed by Top Alliance Biosciences in China, triggered 173 TEAEs in all 40 (100%) participants and 22 drug-related TEAEs in 17 (42.5%) participants [22]. Another fully humanized monoclonal antibody LY-CovMab developed by Shandong BoAn Biotechnology Co., Ltd., triggered 18 drug-related TEAEs reported in 12 subjects (30.0%) [23]. With a Fc mutation design to attenuate ADE activity, JMB2002 demonstrated safety and well tolerated in the similar dose ranges.

The pharmacokinetic parameters C_max_, AUC_0 − t_, AUC_0−∞_ of JMB2002 increased along with dose over a range of 5 to 50 mg/kg, in a similar manner reported previously in the study of Etesevimab and LY-CovMab [22, 23], while no change in elimination half-life (t_1/2_) across dose levels. Therefore, the JMB2002 had similar PK profile with the other antibodies under development. On the other hand, only 1 (2.5%) participant tested positive for ADAs, which was unrelated to the antibody administration. In comparison, 3 (7.5%) participants tested positive for ADA with Etesevimab in the test groups throughout the study, and 5 (12.5%) participants were tested ADA positive caused by LY- CovMab. Therefore, the JMB2002 had similar or lower immunogenicity risk to the similar antibodies under development, correlated with the prediction by in silico analysis and somatic hypermutation rate [17].

In contrast to many NAbs showing low affinity or efficacy to the most recent Omicron variant [15, 17], our in vitro study demonstrated that JMB2002 bound to Omicronspike protein as good as to wild type, in addition to large range of other variants [5]. JMB2002 represents a new class of antibody against the spike trimer, recognizing adistinct epitope on RBD of spike protein from that for previously defined antibodies. It was able to directly inhibit the binding of ACE2 to the Omicron spike trimer and other wide spectrum of variants, and effectively blocked the entry of the Omicron pseudovirus into human ACE2-expressing cells as well as the original SARS-CoV-2 pseudovirus in the neutralization assays [5, 16]. Therefore, JMB2002 have the potential to be effective against the Omicron, which had become the dominant strain in recent months due to its super infectivity, as well as other forth coming variants.

There are several limitations in this study. First, as a phase 1 clinical trial, the sample size is small. Second, participants were recruited from healthy Chinese adults aged 21–45 years old. It remains undetermined whether JMB2002 is safe for high-risk population, such as older adults and those with underlying comorbidities including but not limit to hypertension, diabetes, or obesity [24], or mild-to-moderate COVID-19 patients as the targeted population for the therapeutic purpose. This study did not address the antiviral efficacy in human. Preclinical study in rhesus macaques suggested that there are sufficient prophylactic and therapeutic efficacies of JMB2002 against SARS-CoV-2 infection at 20 mg/kg level, which needs to be directly confirmed in patients.

## Conclusion

In conclusion, the result of this study confirmed the safety and tolerability of JMB2002 in healthy participants, and warrants the phase II clinical trial to explore its efficacy and safety in patients with novel coronavirus pneumonia.

## Data Availability

The datasets used and/or analyzed during the current study are available from the corresponding author on reasonable request.
